# Impact of mass-screening on tuberculosis incidence in a prospective cohort of Brazilian prisoners

**DOI:** 10.1186/s12879-016-1868-5

**Published:** 2016-10-03

**Authors:** Dayse Sanchez Guimarães Paião, Everton Ferreira Lemos, Andrea da Silva Santos Carbone, Renata Viebrantz Enne Sgarbi, Alexandre Laranjeira Junior, Fellipe Matos da Silva, Letícia Marques Brandão, Luciana Squarizi dos Santos, Vaneli Silva Martins, Simone Simionatto, Ana Rita Coimbra Motta-Castro, Maurício Antônio Pompílio, Juliana Urrego, Albert Icksang Ko, Jason Randolph Andrews, Julio Croda

**Affiliations:** 1 Faculty of Health Sciences, Federal University of Grande Dourados, Dourados, Brazil; 2 School of Medicine, Federal University of Mato Grosso do Sul, Campo Grande, Brazil; 3 Faculty of Ambiental and Biological Sciences, Federal University of Grande Dourados, Dourados, Brazil; 4 Department of Biochemical Pharmacy, Federal University of Mato Grosso do Sul, Campo Grande, Brazil; 5 Oswaldo Cruz Foundation, Campo Grande, Brazil; 6 Department of Epidemiology of Microbial Diseases, Yale School of Public Health, New Haven, CT USA; 7 Oswaldo Cruz Foundation, Salvador, Brazil; 8 Division of Infectious Diseases and Geographic Medicine, Stanford School of Medicine, Palo Alto, CA USA

**Keywords:** Tuberculosis, LTBI, Mass screening, Prisons, Case finding, Brazil, Cohort

## Abstract

**Background:**

Globally, prison inmates are a high-risk population for tuberculosis (TB), but the specific drivers of disease and impact of mass screening interventions are poorly understood.

**Methods:**

We performed a prospective cohort study to characterize the incidence and risk factors for tuberculosis infection and disease in 12 Brazilian prisons, and to investigate the effect of mass screening on subsequent disease risk. After recruiting a stratified random sample of inmates, we administered a questionnaire to ascertain symptoms and potential risk factors for tuberculosis; performed tuberculin skin testing (TST); collected sera for HIV testing; and obtained two sputum samples for smear microscopy and culture, from participants reporting a cough of any duration. We repeated the questionnaire and all tests for inmates who remained incarcerated after 1 year. TST conversion was defined as TST ≥10 mm and an induration increase of at least 6 mm in an individual with a baseline TST <10 mm. Cox proportional hazard models were performed to identify risk factors associated with active TB. To evaluate the impact of screening on subsequent risk of disease, we compared TB notifications over one year among individuals randomized to screening for active TB with those not randomized to screening.

**Results:**

Among 3,771 inmates recruited, 3,380 (89.6 %) were enrolled in the study, and 1,422 remained incarcerated after one year. Among 1,350 inmates (94.9 %) with paired TSTs at baseline and one-year follow-up, 25.7 % (272/1060) converted to positive. Among those incarcerated for the year, 10 (0.7 %) had TB at baseline and 25 (1.8 %) were diagnosed with TB over the subsequent year. Cases identified through active screening were less likely to be smear­positive than passively detected cases (10.0 % vs 50.9 %; *p* < 0.01), suggesting early case detection. However, there was no reduction in subsequent disease among individuals actively screened versus those not screened (1.3 % vs 1.7 %; *p* = 0.88). Drug use during the year (AHR 3.22; 95 % CI 1.05–9.89) and knows somebody with TB were (AHR 2.86; 95 % CI 1.01–8.10) associated with active TB during one year of follow up

**Conclusions:**

Mass screening in twelve Brazilian prisons did not reduce risk of subsequent disease in twelve Brazilian prisons, likely due to an extremely high force of infection. New approaches are needed to control TB in this high-transmission setting.

## Background

Globally, incarcerated populations have among the highest tuberculosis (TB) notification rates, frequently in excess of 20 times the rates of their corresponding non-incarcerated communities [1]. Brazil has the world’s 4^th^ largest prisoner population, which grew by 86 % (335,410–622,202) between 2007 and 2015 (http://www.prisonstudies.org/highest-to-lowest/prison-population-total). While TB incidence is declining in community settings across Brazil, it has risen by nearly 40 % among incarcerated population over the past seven years, representing 8 % of approximately 70,000 TB cases reported annually to the Ministry of Health [2]. Moreover, a number of cross-sectional studies have demonstrated a high prevalence of undiagnosed TB among prisoners in Brazil [1, 3–6].

Prisons are optimal environments for TB transmission since they bring together individuals with high rates of tobacco, alcohol, and drug use, and limited access to healthcare and TB diagnostics, into crowded, poorly ventilated cells for 16–20 h a day [7, 8]. Determining which interventions would be most effective and efficient in reducing the burden of TB in such settings is difficult due to the lack of data.

The World Health Organization (WHO) and Brazil’s Ministry of Health recommend screening for active TB upon initial entry into prisons and then annually. Yet, to date Brazilian prisons have not implemented mass screening recommendations since chest x-radiography or laboratory facilities are not available. Cross-sectional studies have demonstrated a high yield of annual screening for active TB among prisoners on a one-time basis, but the yield of this approach over time and impact on subsequent TB incidence remains uncertain [1, 3, 4].

In a previous model-based analysis, we projected a modest reduction (8.3 %) in tuberculosis infection risk following a 25 % reduction in time to TB diagnosis in 3 Brazilian prisons [7]. We hypothesized that the annual mass screening with smear and culture could avert even more transmission and thereby reduce TB incidence in this setting. To address these questions, we designed a prospective cohort study to evaluate the impact of annual screening and assess TB infection and disease rates in 12 Brazilian prisons.

## Methods

### Study location, population and study design

Brazil has a population of 200.4 million and 622,202 prisoners (incarceration rate of 311.1 per 100,000 population). Mato Grosso do Sul state, located in Midwestern Brazil, has a population of 2.5 million with 14,904 prisoners, representing the highest national incarceration rate (596.2 per 100,000 population) [9]. The penal system is comprised of “closed” and “open” subsets to segregate high and low-risk offenders (the latter of whom are allowed to leave the prison during daytime hours); 9,913 inmates in 22 penitentiaries comprise the total closed subset. From January 2013 to December 2014, we conducted a prospective cohort study in 8 male and 4 female prisons in the cities of Campo Grande, Corumbá, Dourados, Ponta Porã, and Três Lagoas. The study population, 7,221 inmates, represents 59 % of the total state inmate population and 73 % of inmates in the closed subset. Of the 12 study prisons, 8 male prisons included 6,552 inmates and 4 female prisons included 669 inmates.

Prior to the study, none of these 12 selected prisons implemented the entrance and annual mass TB screening recommended by WHO [10], and all TB cases were detected passively by inmates seeking medical consultation based on their symptoms; clinicians used clinical assessment, smear and culture to make diagnoses. We conducted a prospective study of active tuberculosis screening in the study prisons.

### Sample size calculation

Prisoners who were 18 years of age and who consented to participate were recruited for the study. Proportional stratified sampling was performed by using each prison as a unit of randomization, as reported previously [8]. Screening for tuberculosis (reported here) was performed alongside parallel screening studies for HIV, Hepatitis B, Hepatitis C, and syphilis. The sample size was calculated based on the expected prevalence of HIV, as this was the lowest prevalence condition for the overall study. We assumed 2 % prevalence of HIV with an error of +/−1 %, power of 80 % and alpha-type error of 5 %. The study population is 7,221 prisoners, and the sample size was 3,159 prisoners. We added 20 % more individuals to account for anticipated loss due to refusal to participate, for a total of 3,771 prisoners.

### Study procedures

We administered a questionnaire and tuberculin skin test (TST) (PPD RT23, Staten Serum Institute, Copenhagen) to all patients, and performed sputum smear microscopy and culture for all participants reporting cough. The variables obtained during the interview included age, sex, marital status, educational attainment, drug use over the last year, history of sexually transmitted infections (STIs), smoking, tuberculosis, self-reported mental illness and diabetes mellitus, knows someone with TB and previous incarceration. The participant’s race/ethnicity (i.e., white, black, indigenous, Asian or mixed) was self-reported.

We scored clinical symptoms and signs according to WHO guidelines: cough lasting for more than two weeks (2 points), expectoration (2 points), chest pain (1 point), weight loss in the last three months (1 point), and a recent loss of appetite (1 point). A score equal to or greater than 5 points indicate a high probability of active disease [10–12].

One sputum sample was collected on the spot and a second sputum sample was collected the following morning. Serum samples of participants were initially screened with a commercial enzyme linked immunosorbent assay (ELISA) for detection of antibodies against HIV-1 and HIV-2 (Murex HIV-1.2.0, DiaSorin, Italy). All positive and indeterminate specimens were confirmed by Western blot assay (Novopath HIV-I, Immunoblot, BioRad) [8]. TST positivity was defined as an induration of ≥10 mm (≥5 mm for HIV-infected individuals), and TST conversion was defined as TST ≥10 mm and an induration increase of at least 6 mm in an individual with a baseline TST <10 mm [10]. In HIV-positive individuals, TST conversion was defined as an induration increase of at least 6 mm in an individual with a baseline TST <5 mm. Brazilian Ministry of Health (MoH) recommends IPT only for HIV-infected inmates with positive TST.

After one year, we returned and administered a second questionnaire and TST, and again performed smear microscopy and culture for all participants reporting cough. To calculate the yield of smear, individuals with positive sputum smear and/or culture in the first and second screening were included in the active screening group. Additionally, we reviewed prison medical records and the National Notifiable Disease database (Sistema de Informação de Agravos de Notificação National, SINAN) to identify TB cases occurring between the study initiation and conclusion. TB cases identified during these period were included in the passive screening group. All questionnaires were entered twice into the Research Electronic Data Capture database (REDCap, secure online database). SAS version 9.2 (SAS Institute, Cary, NC, USA) was used to analyze bivariable and multivariable models.

### Analytic approach

Tuberculosis cases were considered “actively detected” if they were detected by diagnostics performed during mass screening, conducted at baseline and after one year. Cases were considered “passively detected” if they were detected by routine presentation for medical care between the two mass screening rounds; there was no overlap between the two forms of diagnosis. The “active screening” group was the group of inmates enrolled in the study who were actively screened at baseline and one year follow up. Cumulative incidence of active TB was calculated using all incident cases notified by SINAN between the two screening periods divided by all the prisoners who remained one year in the same prison in the two groups. We used survival curves and log-rank test to compare the cumulative incidences of active tuberculosis among the group who underwent annual screening with the group of individuals who did not undergo annual screening. Cox proportional hazards models were used to estimate crude (CHR) and adjusted hazards ratios (AHR) for active TB. Variables were included in the model if they reached a significance level of *p* < 0.20 by log-rank test, which was then trimmed using stepwise backward selection. Statistical significance was determined at a *p* value of <0.05.

### Ethical issues

All eligible participants provided written informed consent prior to study participation. The study was approved by the Research Ethics Committee at the Federal University of Grande Dourados (Number 793,740). All LTBI in HIV-positive individuals and active TB cases identified during the screening underwent medical consultation after the test results and were referred for free tuberculosis treatment or preventive therapy.

## Results

**Fig. 1:**
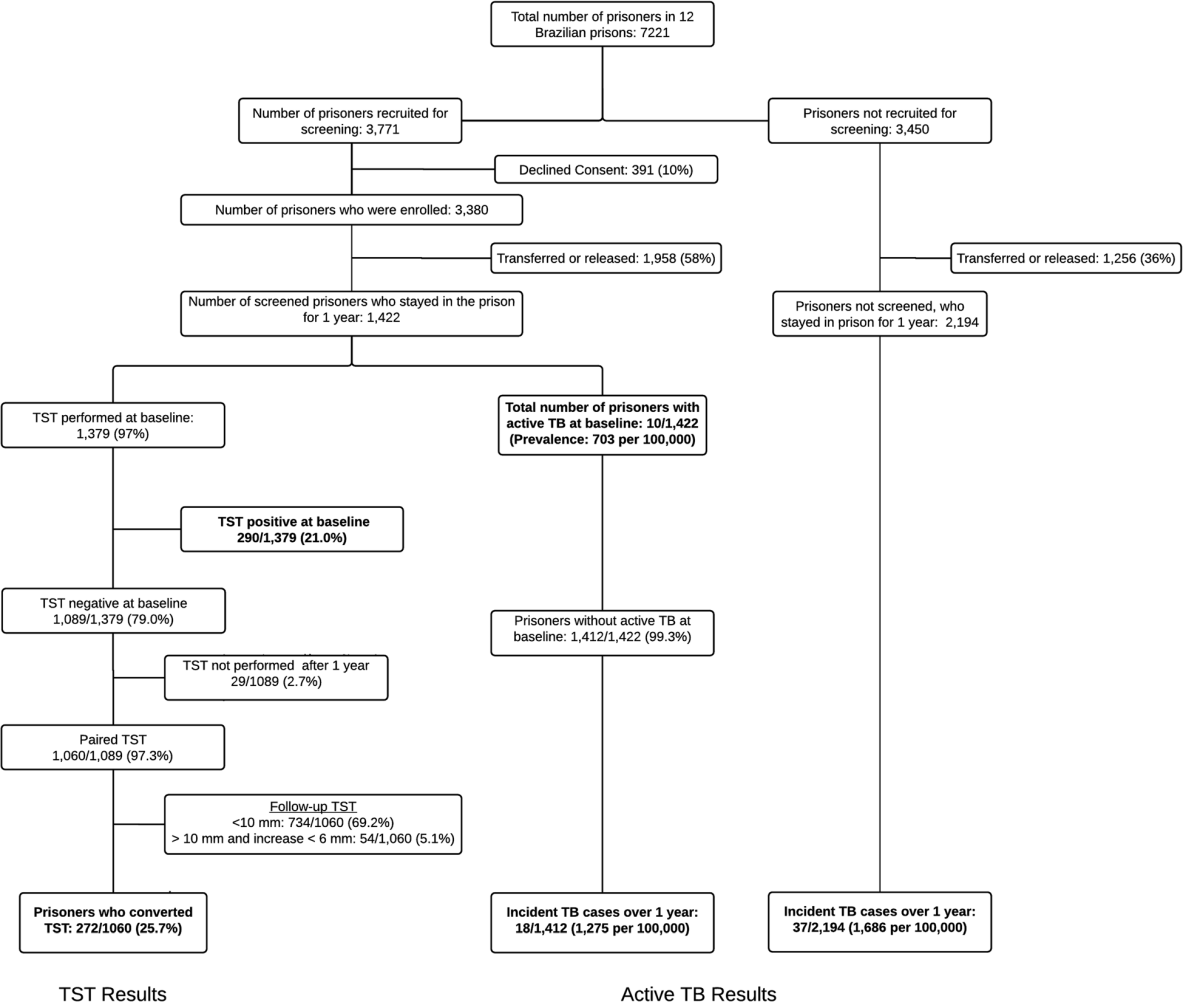
Flow chart of the study enrollment and screening process for active and latent tuberculosis (TB)

From 7,221 inmates in the 12 prisons, we enrolled 3,380 inmates for the study. The results of initial screening were previously reported [8]. Among these, 1,422 remained incarcerated in the same prison after 1 year (Fig. 1); this subset comprises the prospective cohort in whom TST conversions and TB incidence were assessed. The majority of study participants were men (87 %) and mean age was 33 years old (SD: ±10 years, range: 18–80 years). Previous incarceration was reported among 61 % of participants, and 45 % had less than 4 years of schooling. We identified 18 (1.3 %) inmates with positive HIV serology. At baseline, 66 % reported at least 1 TB symptom as defined by the WHO and 23 % had productive cough (Table 1). Among 1,422 inmates followed for one year, baseline latent and active TB prevalence were 21 % (95 % CI: 19–24 %) and 0.7 % (95 % CI: 0.3–1.3 %), respectively (Fig. 1).

**Table 1:** Risk factors associated with incident cases of active tuberculosis (TB) among inmates from 12 Brazilian prisons during one year of follow-up (*N* = 1,422)

Variables	N (%)	Active TB			
		Crude hazard ratio	*p*-value	Adjusted hazard ratio	*p*-value
Age (mean ± SD)	32.6 ± 9.7	0.99 (0.95–1.04)^a^	0.821		
Sex (Male)	1235 (86.8)	2.63 (0.35–19.73)	0.348		
Race					
White	463 (33.9)	1.0			
Mixed	685 (50.2)	0.94 (0.30–2.98)	0.924		
Black	170 (12.5)	3.30 (1.01–10.83)	0.048^b^		
Indigenous	19 (1.4)	-			
Asian	28 (2.1)	-			
Reported any WHO TB symptom at baseline	934 (65.7)	2.63 (0.76–9.09)	0.126^b^		
Reported productive cough at baseline	325 (22.9)	2.25 (0.87–5.80)	0.094^b^		
Less than 4 year of schooling	772 (55.5)	1.63 (0.61–4.36)	0.323		
Diabetes Mellitus	0 (0.0)	-	-		
Current Smoker	730 (51.8)	2.47 (0.88–6.94)	0.085^b^		
HIV positive	18 (1.3)	-	-		
Drug use over the last year	739 (52.0)	3.91 (1.29–11.87)	0.016^b^	3.22 (1.05–9.89)	0.042
Previous incarceration	860 (60.8)	1.03 (0.40–2.66)	0.951		
TST positive	290 (21.0)	1.74 (0.60–5.00)	0.306		
Previous TB	92 (6.6)	3.01 (0.90–10.70)	0.074^b^		
Knows someone with TB	607 (43.5)	3.45 (1.24–9.73)	0.018^b^	2.86 (1.01–8.10)	0.049

^a^Per 1-year increase

^b^Variables initially included in the multivariate model, which was trimmed through backward selection

A second TST was performed in 1,379 (97 %) inmates who remained incarcerated at one year. Among 1,089 participants with a negative TST at baseline, 1060 had a TST performed at one year, and 272 (25.7 %, 95 % CI 22.7–28.9 %) converted. TST conversion was higher in male compared to female prisoners (28 % versus 10 %; *p* < 0.01), while TB incidence was non-significantly higher (1.39 % versus 0.53 %; *p* = 0.68) (Table 2). No difference in TST conversion rates was observed when comparing HIV-uninfected and HIV-infected prisoners (26 % versus 25 %, *p* = 0.95).

**Table 2:** Tuberculin skin test (TST) conversions and tuberculosis (TB) incidence in 8 male and 4 female Brazilian prisons (*N* = 1,422)

Variables	Prisons		
	Male	Female	Total
Capacity	2,469	451	2,920
Inmate population	6,552	669	7,221
Individuals enrolled at baseline	2,861	519	3,380
Individuals followed for 1 year	1,235	187	1,422
TST-negative subjects	905	155	1,060
TST converted subjects	256	16	272
TST conversion rate, %^a^	28 (25–32)	10 (6–17)	26 (23–29)
Incident cases reported between screenings	17	1	18
TB cases at 1^st^ screening	10	0	10
TB cases at 2^nd^ screening	7	0	7
TB incidence, %^a,b^	1.39 (0.81–2.22)	0.53 (0.01–2.98)	1.27 (0.76–2.02)

Abbreviations: *TB* tuberculosis; *TST* tuberculin skin test

^a^Percentage and 95 % confidence interval

^b^Cases identified between screenings or at second screening as a proportion of individuals followed for 1 year

During the first year of follow-up, active TB was diagnosed in 18 participants in the active screening group. All tested negative for HIV at baseline and after 1 year. All but 1 case occurred among male inmates, and 9 (36 %) occurred in just one prison, the maximum-security prison in Campo Grande. Seven cases were diagnosed at the time of the second survey (Table 2).

**Fig. 2:**
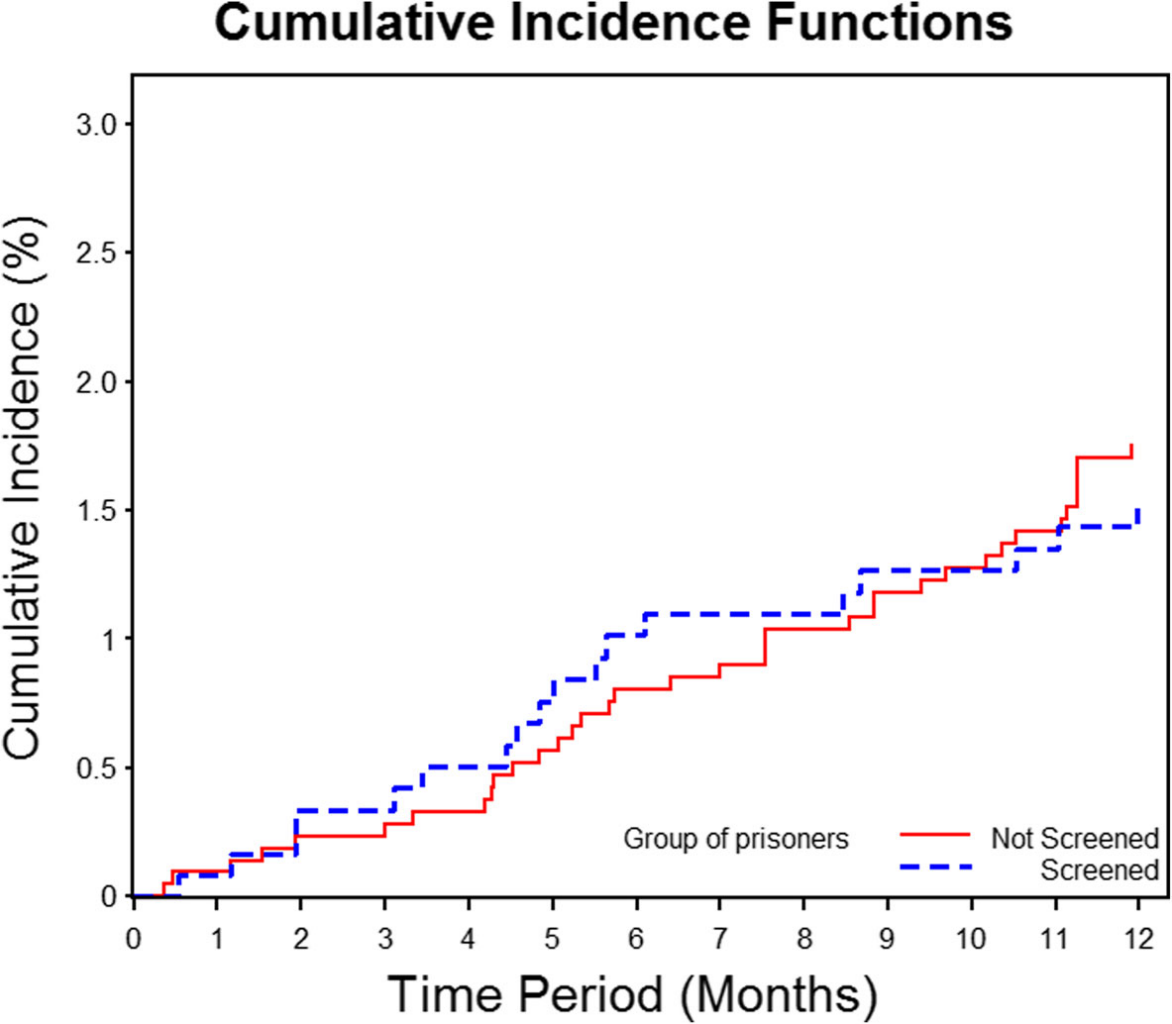
One-year cumulative TB incidence among individuals randomized to the study (actively screened; *N* = 1,422) versus non-randomized (not screened; *N* = 2,194); individuals with active TB at baseline screening and those who left the prison during the first year were excluded

While cases identified through active screening were much less likely to be smear positive than those identified by passive case detection (10.0 % vs 50.9 %; *p* < 0.01), there was no statistical difference in the subsequent cumulative incidence of the group that was actively screened versus those not screened (1.3 % vs 1.7 %; log-rank p value = 0.88) (Fig. 2). Drug susceptibility testing are done by conventional broth-based culture methods using the MGIT 960 system and did not identify any case of multidrug-resistant tuberculosis. Drug use during the year (AHR 3.22; 95 % CI 1.05–9.89) and knows somebody with TB were (AHR 2.86; 95 % CI 1.01–8.10) associated with active TB during one year of follow up (Table 1).

## Discussion

The extraordinarily high incidence of TB infection (25.7 %) and disease (1,275 per 100,000 inmates) indicate a critical need to address TB transmission inside prisons. By comparing TB prevalence with incidence, we estimate an average 6-month duration of disease before diagnosis, death, or release; it is during this window that transmission occurs. While this period may be longer due to underestimation of prevalence, it nevertheless reflects a narrow window for diagnosis, in comparison with community estimates of infectious duration that are often twice as long [13].

The effective contact rate (annual risk of infection divided by prevalence) during this period is extremely high (>36/year), reflecting high rates of transmission and could represents the confluence of several factors: 1) overcrowding; 2) poorly ventilated environments; 3) limited TB diagnostic infrastructure; and 4) an inmate population with low prevalence of latent tuberculosis infection (LTBI) upon initial incarceration, who are at higher risk for infection upon exposure [7, 8, 14–16].

WHO and the Brazilian Ministry of Health recommend screening for TB in prions at entry and annually; however, in practice, this was not being performed in any of the study prisons. In this study, mass screening appeared to identify patients early in the course of disease, as reflected by lower smear positivity rates (10.0 % vs 50.9 % by passive diagnosis); similar findings concerning smear positivity have been demonstrated in community settings comparing active and passive case detection [17]. However, subsequent TB incidence among screened individuals was not significantly reduced. This may be a result of the extremely high force of infection driving new cases. Moreover, the majority (72 %) of TB diagnoses were made passively between the two active screening points. Enhanced screening for active TB, while valuable, may be insufficient to effectively control TB in prisons [8]. Most likely, multiple interventions will be necessary to reduce TB transmission in prisons in resource-poor countries. Emphasis on direct measures to reduce transmission, such as reducing crowding and improving ventilation [7], or reducing disease risk among recently infected inmates, may be needed to control TB under these conditions.

There are two broad classes of proven interventions for averting TB transmission: expedited diagnosis with treatment of infectious cases, and preventive therapy for LTBI. In Alaska (U.S.), for example, vigorous programs for early diagnosis, treatment and isoniazid preventive therapy (IPT) had rapid and dramatic effects on TB incidence and transmission [18, 19]. Currently, the WHO provides a conditional recommendation for LTBI screening and provision of isoniazid preventive therapy (IPT) in prisons in high and upper middle-income countries [20], but the Brazilian MoH does not recommend treating inmates with a positive TST. In contrast, treatment is recommended and has been shown to be effective in controlling the disease in other high-risk populations, such as indigenous groups [21]. Screening for TB at the entry and annually, with provision of IPT for infected individuals, has been effective at reducing TB incidence in the United States [22], but there are few published data on the use of IPT in penal institutions in resource-poor countries. Our finding of low LTBI prevalence at time of prison admission and a high rate of TST conversion after one year suggests that annual TST screening and treatment of LTBI could potentially be an effective intervention in this setting [23].

Although two previous cross-sectional studies have shown that drug use was associated with active TB in prisoners populations [24, 25], this is the first cohort study designed to identify individual risk factors associated with active TB inside the prisons. Drug use over the last year (AHR 3.22; 95 % CI 1.05–9.89) was associated with active TB and drug abuse program need to implement to reduce the incidence of active TB in prisons.

Urrego et al. documented overcrowding and inadequate ventilation in 3 prisons included in this study [7]. In a recent study in prisons in Chile, overcrowding was identified as a key determinant of LTBI among contacts of active TB cases [14]. In our study, female prisons were at 148 % (669/451) capacity, while male prisons were at 265 % (6,552/2,469) capacity (Table 2). The higher TST conversion rates and active TB incidence in male prisons compared with female prisoners may be in part due to greater overcrowding in the former.

Our findings are subject to several limitations. We used the TST conversion to assess tuberculosis infections, which has imperfect sensitivity and specificity, but is the primary modality of latent tuberculosis infection testing in Brazil. To test for active tuberculosis, we performed smear and culture on two sputum specimens. Chest radiography is not available in the prisons, which could enhance screening sensitivity and could potentially decrease TB transmission and incidence. While recommended by Brazilian guidelines, screening for TB upon entry into prisons is not routinely performed. TB incidence in the general population of this state is <40 per 100,000, and we have previously found LTBI prevalence of <10 % at entry to prisons [8], suggesting entry screening may have low yield [26].

At the individual level, we found no impact of screening on subsequent TB diagnosis rates. We enrolled only half (3,380/7,221) of the total prisoner population, and transmission from the unscreened group may have limited any population-level transmission benefits of screening. Achieving higher screening coverage and screening more frequently would likely have a greater impact on subsequent TB incidence in both the screened and non-screened population. A caveat is that the median duration of incarceration is under 2 years in this population, meaning that the inmate population turns over quickly between annual screenings, potentially diminishing the impact of this intervention. An additional consequence of the high turnover rates are that loss to follow-up in prospective cohorts of prisoners are high, as was the case in this study; since we randomly selected inmates for screening, this was unlikely to bias the study findings. We did not collect clinical or demographic data on the inmates who were not enrolled in the screening group; as we randomly selected individuals for recruitment and participation rates were high, the risk for selection bias was low. Additionally, our findings were from the “closed” system, where prisoners are incarcerated continuously, and further work is needed to characterize risks in the “open” system, a transitional environment where prisoners are only housed at night.

## Conclusions

In a network of 12 prisons in central western Brazil, TB infection and disease rates were extraordinarily high. More aggressive interventions—including more frequent screening and use of preventive therapy—may be required to reduce the burden of TB in this high transmission setting. Moreover, the high TB rates found in these prisons not only represent an institutional public health crisis, but also a threat to the control of TB in the broader population, as supported by molecular evidence of spillover in this community [15]. The high rate of incarceration and movement combined with extraordinary infection rates, indicate that prisons can be important reservoirs of TB transmission to the general population. Urgent interventions are needed to address the unimpeded spread of TB in Brazil’s prisons.

## Abbreviations

AHR: adjusted hazard ratio
CHR: crude hazard ratio
CI: confidential interval
CXR: Chest X radiography
ELISA: enzyme-linked immuno sorbent assay, HIV, human immunodeficiency virus
IPT: isoniazid preventive therapy
LTBI: latent tuberculosis infection
MGIT: Mycobacteria Growth Indicator Tube
PPD: purified protein derivative
REDCap: Research Electronic Data Capture database
SD: standard deviation
SINAN: Sistema de Informação de Agravos de Notificação National
TB: tuberculosis
TST: tuberculin skin test
WHO: World Health Organization

## References

[1] Baussano I, Williams BG, Nunn P, Beggiato M, Fedeli U, Scano F (2010). Tuberculosis incidence in prisons: a systematic review. PLoS Med.

[2] Bourdillon P, Croda J, Ko A, Andrews JR. Rapidly Increasing Tuberculosis Burden in Brazilian Prisons Offsets Gains in General Population. Int J Tuberc Lung Dis. 2015;19(12):S436–7.

[3] Kuhleis D, Ribeiro AW, Costa ER, Cafrune PI, Schmid KB, Costa LL, Ribeiro MO, Zaha A, Rossetti ML (2012). Tuberculosis in a southern Brazilian prison. Mem Inst Oswaldo Cruz.

[4] Sanchez A, Massari V, Gerhardt G, Espinola AB, Siriwardana M, Camacho LA, Larouze B (2013). X ray screening at entry and systematic screening for the control of tuberculosis in a highly endemic prison. BMC Public Health.

[5] Adane K, Spigt M, Ferede S, Asmelash T, Abebe M, Dinant GJ (2016). Half of Pulmonary Tuberculosis Cases Were Left Undiagnosed in Prisons of the Tigray Region of Ethiopia: Implications for Tuberculosis Control. PloS One.

[6] Telisinghe L, Fielding KL, Malden JL, Hanifa Y, Churchyard GJ, Grant AD, Charalambous S (2014). High tuberculosis prevalence in a South African prison: the need for routine tuberculosis screening. PloS One.

[7] Urrego J, Ko AI, da Silva Santos Carbone A, Paiao DS, Sgarbi RV, Yeckel CW, Andrews JR, Croda J (2015). The Impact of Ventilation and Early Diagnosis on Tuberculosis Transmission in Brazilian Prisons. Am J Trop Med Hyg.

[8] Carbone Ada S, Paiao DS, Sgarbi RV, Lemos EF, Cazanti RF, Ota MM, Junior AL, Bampi JV, Elias VP, Simionatto S (2015). Active and latent tuberculosis in Brazilian correctional facilities: a cross-sectional study. BMC Infect Dis.

[9] Departamento Penitenciário Nacional MdJ, Brasil. Levantamento Nacional de Informações PenitenciáriasINFOPEN - JUNHO 2014. In: 2014. http://www.justica.gov.br/noticias/mj-divulgara-novo-relatorio-do-infopen-nestaterca-feira/relatorio-depen-versao-web.pdf. Accessed 27 Sep 2016.

[10] Tuberculosis Control in Prisons. World Health Organization. 2000. http://apps.who.int/iris/bitstream/10665/66823/1/WHO_CDS_TB_2000.281.pdf. Acessed 27 Sep 2016.

[11] Aerts A, Habouzit M, Mschiladze L, Malakmadze N, Sadradze N, Menteshashvili O, Portaels F, Sudre P (2000). Pulmonary tuberculosis in prisons of the ex-USSR state Georgia: results of a nation-wide prevalence survey among sentenced inmates. Int J Tuberc Lung Dis.

[12] Sanchez A, Gerhardt G, Natal S, Capone D, Espinola A, Costa W, Pires J, Barreto A, Biondi E, Larouze B (2005). Prevalence of pulmonary tuberculosis and comparative evaluation of screening strategies in a Brazilian prison. Int J Tuberc Lung Dis.

[13] Wood R, Middelkoop K, Myer L, Grant AD, Whitelaw A, Lawn SD, Kaplan G, Huebner R, McIntyre J, Bekker LG (2007). Undiagnosed tuberculosis in a community with high HIV prevalence: implications for tuberculosis control. Am J Respir Crit Care Med.

[14] Aguilera XP, Gonzalez C, Najera-De Ferrari M, Hirmas M, Delgado I, Olea A, Lezaeta L, Montana A, Gonzalez P, Hormazabal JC (2016). Tuberculosis in prisoners and their contacts in Chile: estimating incidence and latent infection. Int J Tuberc Lung Dis.

[15] Dara M, Acosta CD, Melchers NV, Al-Darraji HA, Chorgoliani D, Reyes H, Centis R, Sotgiu G, D'Ambrosio L, Chadha SS (2015). Tuberculosis control in prisons: current situation and research gaps. Int J Infect Dis.

[16] Johnstone-Robertson S, Lawn SD, Welte A, Bekker LG, Wood R (2011). Tuberculosis in a South African prison - a transmission modelling analysis. S Afr Med J.

[17] Eang MT, Satha P, Yadav RP, Morishita F, Nishikiori N, van-Maaren P, Weezenbeek CL (2012). Early detection of tuberculosis through community-based active case finding in Cambodia. BMC Public Health.

[18] Comstock GW, Baum C, Snider DE (1979). Isoniazid prophylaxis among Alaskan Eskimos: a final report of the bethel isoniazid studies. Am Rev Respir Dis.

[19] Comstock GW, Philip RN (1961). Decline of the tuberculosis epidemic in Alaska. Public Health Rep.

[20] Getahun H, Matteelli A, Raviglione M. Guidelines on the management of latent tuberculosis infection. In*.* Edited by Organization WH. 2015. http://apps.who.int/iris/bitstream/10665/136471/1/9789241548908_eng.pdf?ua=1&ua=1. Accessed 27 Sept 2016.

[21] Yuhara LS, Sacchi FP, Croda J (2013). Impact of latent infection treatment in indigenous populations. PloS One.

[22] MacIntyre CR, Kendig N, Kummer L, Birago S, Graham NM (1997). Impact of tuberculosis control measures and crowding on the incidence of tuberculous infection in Maryland prisons. Clin Infect Dis.

[23] Chan PC, Yang CH, Chang LY, Wang KF, Lu BY, Lu CY, Shao PL, Hsueh PR, Fang CT, Huang LM (2012). Latent tuberculosis infection treatment for prison inmates: a randomised controlled trial. Int J Tuberc Lung Dis.

[24] Alavi SM, Bakhtiarinia P, Eghtesad M, Albaji A, Salmanzadeh S (2014). A comparative study on the prevalence and risk factors of tuberculosis among the prisoners in khuzestan, South-west iran. Jundishapur J Microbiol.

[25] Adesokan HK, Cadmus EO, Adeyemi WB, Lawal O, Ogunlade CO, Osman E, Olaleye OD, Cadmus S (2014). Prevalence of previously undetected tuberculosis and underlying risk factors for transmission in a prison setting in Ibadan, south-western Nigeria. Afr J Med Med Sci.

[26] Croda MG, Trajber Z, Lima Rda C, Croda J (2012). Tuberculosis control in a highly endemic indigenous community in Brazil. Trans R Soc Trop Med Hyg.

